# Is the scientific paper still a fraud?

**DOI:** 10.1371/journal.pbio.3003912

**Published:** 2026-08-04

**Authors:** John S. Tregoning

**Affiliations:** Department of Infectious Disease, Imperial College London, London, United Kingdom

## Abstract

How we write scientific papers does not reflect how we do science, masking the human in the process by design. This Perspective invites us to consider the structure of the scientific paper, and the need to celebrate the human in an age when AI threatens to overwhelm us.

## Teaser

How we write scientific papers does not reflect how we do science. Their formal structure infers a pre-ordained linear process rather than reflecting the messy creativity of research. This matters in the AI age because it masks the human in the process.

In 1963, Nobel Prize-winning immunologist Peter Medawar presented a controversial idea on BBC radio, that the scientific paper was a fraud [1]. But he did not mean it in the way you might think. Scientific fraud does happen (both then and now), but he meant something more fundamental about the format of the scientific paper. His argument was that the structure of papers tells a false story about how science is done. Papers state the problem to be investigated, the way it was investigated, and the investigations performed, and arrive at the conclusion(s) drawn. Medawar argued that this structure implies scientific research is purely inductive (drawing general conclusions from specific data), leaving almost no role for the scientist except as a neutral note taker. The scientific paper is a very sanitized version of how research works, but it remains the main method by which we communicate science. In the age of artificial intelligence (AI) are Medawar’s arguments about papers still valid, and what can we learn about how we (humans) do science?

We seldom talk about the craft of science, choosing to focus on the data rather than the journey by which it arose. But underneath that data, there is a process that we should consider. The first step is the hypothesis. In the sterile language of papers, these emerge fully formed. But Medawar stated that most hypotheses “arise by guesswork” [1]. The word guesswork seems removed from the idealized view of scientists applying cold logic at the cutting edge of understanding, bringing it more into the realms of creativity [2]. But it is not a negative statement. I agree with Medawar when he said that, “scientists should not be ashamed to admit […] that hypotheses appear in their minds along uncharted byways of thought” [1]. There is an inception point. A moment before which the idea did not exist. Creativity has a central role in science; Medawar described it as “adventures of the mind” [1]. I would insert the word human before mind. In order to do science, you need imagination. It is what makes us scientists and it is ultimately the most rewarding part of the job. AI cannot do this. It is an important tool, especially with regards large datasets that the human brain cannot process, but it cannot imagine.

Having come up with the hypothesis, we then need to be selective about the studies undertaken. Papers, as written, suggest a pre-ordained path, but the journey is necessarily messier. They do not reflect the untaken roads, but those roads less travelled are just as important as those taken, and the human scientist has a critical role in the whole process, drawing on experience (and sometimes luck) to direct the study.

Medawar observed that nearly “all scientific work starts with some expectation about the outcome” [1]. In other words, we mainly do experiments when we suspect we know the answer. This rings true. It is reflected in the minimum traction for the publication of negative data: when you have limited resources (time and/or money), the pull to tell positive stories is far greater than that to explore negative ones. The publish-or-perish pressure shapes the scientific agenda. There are ways around this to set yourself up to reduce so-called “failure.” My favorite types of experiments are those in which results can take a branching path; the worst kind are those where you need a certain answer to complete the narrative you are building. These are often the ones that a reviewer/principal investigator requests to finish up a piece of work: that dreaded “one last experiment,” so crushing when they fail. There are, of course, exceptions, and one wonders what Medawar would have made of hypothesis-free “omics” approaches. But even these are undertaken with some expectation that they will generate informative data about the condition being measured.

Building from the hypothesis, scientific papers tell a story about the order in which the research was allegedly undertaken. And again, the reported progression from data to narrative is not always true. Often, the data presented in the first figure was the last experiment to be performed. Sometimes, data comes from independent studies undertaken by different people at different times. Doing one thing *now* can unlock ideas from the past. But we then have to put this into some form of narrative. This is not getting easier, especially in the days of big data, where you ultimately have to select one gene/protein/metabolite/bacteria from your “ome” to dissect further. In fact, one of the reasons I revisited Medawar’s idea [1] is because a paper we are currently writing does not fit the standard reductionist approach—in the end biology is not reductionist, it is complex, replete with redundancy. This is not to detract from the narrative element of science, fitting the puzzle together requires human input.

Papers, as usually written, remove the agency of the authors. Writing in a uniform “scientific” voice often requires masking any and all personal viewpoints. The formal voice also flattens the prose, removing any of the excitement the researcher feels about the research [3]. In some ways, science is behind other academic disciplines in this regard. History, for example, pays attention to the historian and how their perspective affects the interpretation of the material. This is rarely discussed in science. But we, the authors, make choices about the paths we follow and the stories we choose to tell with the data collected along those paths. The story is, in turn, constrained and shaped by external “contributors;” the reviewers and editors who wish to see more or less of a particular aspect of the story, thereby shaping the interpretation. This current article you are reading is the product of a conversation between myself and the editor Nonia Pariente and her team; and is much improved for it. We are better served as authors when editors edit, giving clear guidance of what works and what does not, rather than acting as gatekeepers. And I encourage you to have these types of conversations.

So was Medawar right and is he still right? In many ways, yes, he was. The paper incorrectly reflects scientific research. As identified by Howitt and Wilson in a previous response to Medawar, the structure can give students a false picture of the research process [4]. It also affects the whole endeavor of science. In an age where we risk being overwhelmed by AI slop, we need to think about the process as much as the product. There have been many attempts to change the peer-review/publication model, for example, pre-prints, Review Commons and eLife’s new publishing model. But the scientific paper remains the same regardless of whether it is pre-printed, open access or pay-per-view. In the end, the whole scientific endeavor is about communicating your findings. Papers are the accepted unit and the likelihood that this will change seems low. But we should be aware of their limitations and supplement them with other communication forms from across the broader continuum of conferences, blogs, and (whisper it quietly) social media. By doing this, we can talk about the process, the people involved, and the resulting science; who knows, by doing this you might even engage a broader audience.

Ultimately, the scientific paper is still a fraud, because it masks the humans in the process. We need to remind ourselves that the paper is just the external manifestation of our research; put into a common form to share with others. The scientific thought that goes into it is the important part. The innovation, intuition, imagination and, yes, guesswork all matter. Most of all, the human in the process matters.

## Abbreviation:

AI: artificial intelligence
